# Sex as Boys’ Fame, But Girls’ Shame: Adversarial Adolescent Gender Roles and Gender-based Violence in Guyana

**DOI:** 10.1177/08862605211043585

**Published:** 2021-09-11

**Authors:** Ruth Rodney, Denise Gastaldo, D. Alissa Trotz, Claire V. Crooks

**Affiliations:** 1 York University, Toronto, ON, Canada; 2 University of Toronto, ON, Canada; 3 Western University, ON, Canada

**Keywords:** dating violence, adolescents, Caribbean, gender roles, violence prevention

## Abstract

Gender-based violence (GBV) is a significant issue for youth in Guyana, particularly among young women. Yet, discussions about sex, dating, and violence rarely occur at the community level. To understand the heightened risk for GBV with youth in Guyana, we utilized a critical qualitative design to explore adolescent dating violence with adolescents (14–16 years old), parents, and school officials in a public secondary school in Guyana. In total, 36 racially and religiously diverse participants from low to middle-income households participated in focus groups (*n* = 30) and interviews (*n* = 6). Discussions centered on dating in adolescence; community awareness of dating violence; gender, racialization, and class in relation to dating violence; and dating violence prevention in schools and family settings. Our results revealed that heteronormative, adversarial gender roles in Guyana are enacted in adolescent relationships in ways that contribute to violence. Two important factors emerged in relation to femininity: female respectability related to sexuality; and the relationship between clothing, sexuality, and social class. Masculinity for adolescent boys was centered on reproducing normative assumptions about femininity and explaining the use of violence through pathologizing race. Participants were also asked to identify gender roles that adolescent boys and girls should embody in relationships, which revealed possibilities for overcoming adversarial roles in relationships. We propose that adolescent GBV prevention initiatives consider long-standing and deeply embedded ideas within gender norms that are connected to sexuality, class, and race. Without accounting for these systemic factors, GBV prevention initiatives and programs may inadvertently perpetuate traditional definitions of masculinity and femininity that contribute to violence.

## Introduction

The most recent report on intimate partner violence (IPV) in Guyana indicates that within the 12 months prior to the report, the risk of experiencing physical and/or sexual violence was highest among younger women (15–24 years old) (Contreras-Urbina et al., 2019). Further compounding the issue is high rates of violence against women in Guyana. At 55% Guyana is well above the global average of 30% of women experiencing physical and/or sexual violence by an intimate partner in their lifetime (Contreras-Urbina et al., 2019; García-Moreno et al., 2013). These statistics align with research globally that indicates younger age can increase the risk of perpetration and victimization of IPV along with lower levels of education and socioeconomic status (Abramsky et al., 2011; Capaldi et al., 2012). Adding to the complexity of this issue, dating during adolescence is not widely accepted in Guyanese culture, which limits a robust public conversation on this issue and therefore the understanding of IPV during adolescence (Rodney, 2017).

To decrease the heightened risk of violence for youth in Guyana, we believe it is imperative to confront this public health crisis through open community dialogue, focusing specifically on longstanding factors that contribute to the acceptability of gender-based violence (GBV). Our first step was to consider the historical context, recognizing that people in Guyana share a history of intense and repeated violence that began at the point of European entry into the Caribbean (Higman, 2010). Guyana’s racially diverse population was created through Indigenous dispossession, transatlantic slavery, and indentureship, with enslaved Africans and indentured laborers from India, China and Portugal brought to Guyana as labor for plantation economies (Haynes, 2012; Higman, 2010). These histories have created deep and abiding social hierarchies, colonial legacies that shape how racially diverse Guyanese people interact today.

Institutional discourses and social practices that occur, for instance, within families and the educational system, have (re)produced colonial inequities, such as heteronormative, patriarchal, and racialized assumptions about family structure, particularly in relation to women-led single-parent households and the legitimacy of intimate relationships based on the union being categorized as a marriage or not (Robinson, 2013; Trotz, 2003). This historical lens supplies a frame to understand the specificity of heterosexual relationships within the Caribbean region and the importance of an intersectional approach that can attend to multiple systemic factors. This background provides an important context to understand and more effectively address contemporary forms of violence in the Caribbean region. Specifically, there is a dearth of information on adolescent dating violence in a Guyanese context because dating during adolescence is not widely accepted. Consequently, experiences that arise from dating relationships are not discussed openly within community settings, even if at times they are discussed within the familial home. Our study addresses this gap in knowledge guided by the following research question: How are dating violence and its prevention in adolescence perceived in Guyana? To our knowledge, there is no other study that has examined adolescent dating violence with these community groups (parents, teachers, and adolescents) to consider how dating violence is socially reproduced in Guyana. In this article, we address elements important for this understanding. Specifically, we discuss how respectability and reputation, concepts often considered when analyzing gender dynamics in adults in a Caribbean context (Besson, 1993; Green, 2006; Wilson, 1995), can be used to inform our understanding of femininity in adolescent girls and masculinity in adolescent boys. Secondly, we address contextual factors that impact the understanding of gender roles and violence among adolescents in Guyana.

## Literature Review

Over the last decade, there have been several studies and projects focused on domestic violence prevention in Guyana and some of these have targeted adolescents (Help and Shelter, 2011; Jackson & Kissoon, 2010; Mancey, 2008; UNICEF, 2013). Some NGO reports included youth, recognizing that men and boys should be educated to understand that abusive behavior is not needed to define masculinity (Mancey & Kissoon, 2010). Other projects aimed at reducing GBV by increasing awareness of violence against women, girls, and children (Help and Shelter, 2011). However, most of these studies have tended to focus on domestic violence as a relationship experience that occurs in adulthood, thus eliding consideration of whether and how it is experienced by youth. In a project focused on domestic violence prevention, adolescents defined gender roles that reflected masculinity as men having financial and decision-making responsibilities as well as fathering a child, and femininity as women displaying loyalty and passivity as well as mothering a child (Mancey, 2008). This understanding of gender roles pervades patriarchal and heteronormative societies globally. Given the explicit focus on parenting and child-rearing, it is unclear how adolescents understand their masculinity and femininity outside of these aspects and even if violence in adolescent relationships is recognized as such, if these components are not present. In another example, after adolescents completed a program focusing on reducing GBV, it was indicated that they were able to identify abuse within the home, among family members, and from teachers, but there was no indication that violence in dating relationships among peers was recognized (Help and Shelter, 2011).

Yet, a common thread throughout Guyanese domestic violence studies/projects has been a discussion of how heteronormative gender roles contribute to GBV (Help and Shelter, 2011; Jackson & Kissoon, 2010; Mancey, 2008; Mancey & Kissoon, 2010). Similarly, studies from other locations have clearly indicated that inequitable gender norms are significantly connected to the acceptance of IPV (Endut et al., 2020; Fine et al., 2019; Flood & Pease, 2009; Herrero et al., 2017; Jakobsen, 2014; Ulloa et al., 2008). In particular, a body of literature has focused on the role of normative gender roles in adolescent dating violence elsewhere around the globe (Lichter & McCloskey, 2004; Reyes et al., 2016; Shen et al., 2012). Some studies have shown that while traditional gender roles have been found to contribute to dating violence, they do not have the same effect on different forms of violence (i.e., sexual, physical, and psychological). Sears et al. (2007) and Shen et al. (2012) found a relation between traditional gender roles and sexual violence, but not physical violence. In addition, some researchers have argued that traditional gender roles can act as a protective factor against GBV, as males assumed the role of protector (Poteat et al., 2011; Reyes et al., 2016).

Even though traditional gender roles have been a main area of focus to understand GBV, examining the acceptability of violence alongside traditional gender roles is important to explain patterns in adolescent dating violence. Reyes et al. (2016) indicate that some adolescent boys can adhere to traditional gender norms and not engage in dating violence because they understand violence to be inherently wrong. Studies that focus on intergenerational violence illustrate that some adolescents who are exposed to domestic violence in their familial home are at greater risk of perpetrating and/or being a victim of dating violence (Abramsky et al., 2011; Capaldi et al., 2012; Flood & Pease, 2009; WHO, 2009; Wolfe et al., 2009), where some research has found that adolescent girls were not more susceptible to accepting physical violence even though they had been exposed to familial violence, while boys who had experienced childhood familial victimization had strong attitudes accepting physical violence against women and girls (Debowska et al., 2017). Therefore, the impact of violence on youth can vary based on gender and gendered assumptions (Debowska et al., 2017; Ulloa et al., 2008).

International literature on GBV reveals that the understanding of traditional gender norms in relation to the use of violence must be addressed, yet there are gaps in our knowledge. First, few studies have focused specifically on youth in the Caribbean. Those that did have documented prevalence of IPV, attitudes towards IPV, and violence prevention initiatives (Alexander et al., 2014; Debowska et al., 2017; Le Franc et al., 2008). However, to our knowledge, no studies have examined *how* traditional gender norms are understood from the perspectives of adolescents and their surrounding communities (educational, familial, residential) and further, how these common-sense understandings can contribute to GBV in adolescence.

Research has also acknowledged that further studies are needed to understand how gender norms are lived out and maintained within adolescent communities (Reyes et al., 2016). Secondly, the analysis in several studies does not delve into the systemic factors that inform the understanding of gender roles (McCauley et al., 2013; Reyes et al., 2016). This is particularly important because understanding how gender norms are socially constructed and understood in adolescence is necessary to reimagine how equitable gender roles can be enacted by youth (McCauley et al., 2013; Reyes et al., 2016).

Specifically, few studies seek to understand how race, as a socially constructed characteristic, impacts the understanding of gender roles and dating violence in adolescence. Stueve and O’Donnell (2008) and Savasuk-Lukston (2019) address racial discrimination and how it impacts dating violence in American society. The context of race has been considered in predominantly American studies (Savasuk-Luxton et al., 2018; Storer et al., 2020; Stueve & O’Donnell, 2008) and in adult Guyanese populations with regard to gender and violence (Trotz, 2003, 2004), but there is minimal examination of race, gender roles, and violence among Guyanese adolescent populations.

## Methodology

In this qualitative study, our aim was to examine adolescent dating violence from the perspectives of adolescents, parents, and teachers in a public secondary school located in Guyana’s capital city, Georgetown. We situate our understanding of dating violence with recognition that discourses on dating and dating violence do not occur in silo. They are informed by interactions with the communities that surround adolescents and therefore bear significant responsibility for the socialization of Guyanese youth into gendered life. Consequently, it was important to include parents and teachers in this study as parents play a key role in shaping how adolescents understand and respond to violence and teachers have significant interactions with adolescents due to the time spent in school.

Considering that this topic is not commonly discussed, we used a critical exploratory qualitative design. A critical exploratory methodology combines a critical theory analytical lens with exploratory methodologies. Approaching adolescent dating violence through a critical lens, meant that we critiqued and challenged the current understanding of factors that contribute to adolescent dating violence by asking who has power, how is it negotiated and what structures in society reinforce the distributions of power (Merriam, 2009). Our theoretical lens not only informed our analysis but also guided the decisions we made throughout data collection to ensure theoretical and methodological congruence. Exploratory designs are typically used when there is a lack of information on a topic or when a topic is being explored through a different lens (Hesser-Biber & Leavy, 2011). Given that no study of its kind had been completed, an exploratory design was ideal to examine the complexity of adolescent dating violence in Guyana.

While the research team resides outside of Guyana, the first author is of Guyanese heritage along with another research member. Given that this topic is rarely discussed publicly in Guyana we engaged in pre-fieldwork a year before data collection occurred. We saw this as a necessary step to build trust and support for our work and slowly establish working relationships. The first author met with local stakeholders who focus on domestic violence in Guyana and the Chief Education Officer for the Ministry of Education at that time. These meetings resulted in the creation of an in-country advisory board that offered guidance and support throughout the duration of this study. This study was reviewed and approved by the University of Toronto Research Ethics Board and authorized by the Ministry of Education in Guyana.

### Recruitment and Participants

In total, we recruited 36 participants from racially and religiously diverse low to middle-income families in the study; school officials (*n* = 4) and parents (*n* = 2) participated in interviews and teachers (*n* = 8), parents (*n* = 7), boys (*n* = 5) and girls (*n* = 10) participated in focus groups. All participants who agreed to be interviewed or participate in a focus group were included in the study, except for the boys focus group. While eight boys agreed to participate, five showed up to focus group discussions. While eight boys returned invitation forms indicating they would participate, five showed up on the days for the group discussions. Teacher and parent focus groups included women and men, whereas adolescent focus groups were separated by gender. Parents and students from different households were recruited for focus group discussions by sending information letters home with two Form 4 (Grade 10) and two Form 5 (Grade 11) classes.

Racially diverse students between the ages of 14 to 16 years old (*n* = 10 females/*n* = 5 males) and parents of adolescents between 14 to 16 years old (*n* = 5 females/*n* = 2 males) participated in focus groups. Snowball sampling was used to obtain the six key informant interviews. Adults received $3,700 GYD ($20CAD) and adolescents received $1,700GYD ($10CAD) as compensation for the time dedicated to this study. Informed consent was obtained for adults and youth prior to interviews and focus group discussions. Data were generated over a two-month period.

### Methods: Interviews and Focus Groups

Patton’s (2002) style guide informed the creation of semi-structured interview guides. Interviews and focus group guides posed questions about dating, dating violence, and dating violence prevention (refer to Tables 1 and 2). A summary of the overarching themes with examples of some questions posed to participants is provided in both Tables 1 and 2. Interview and focus group guides were tailored to the participant group interviewed and questions evolved based on the conversations that occurred within the interview or group discussions.

**Table 1.** Summary of Focus Group Questions for Adolescent Girls and Boys.

Dating in adolescence	What is the difference between being a friend and dating someone? What do you call it? Does dating involve having sex or not?
Dating violence (Questions relating to newspaper article)	How would you describe the relationships in these stories? What are some of the things that stand out to you in these stories?
Community awareness	How is violence viewed in the community? Is violence something that is talked about? If so, where and when is it mostly discussed?
Gender (Questions in relation to newspaper articles)	If this story were the other way around, and Angela or Sheniza was the attacker, would the story be any different? How so?
Ethnicity and class (Questions in relation to newspaper articles)	Sheniza and Angela come from different ethnic backgrounds, does this impact how you see their stories? Is it different or the same if you go through something like this and you’re Afro-Guyanese or Indo-Guyanese? Does living in one part of Guyana as opposed to another effect being in situations that are violent?
Prevention of dating violence in adolescence	Can you identify any activities, programs, or resources that address dating violence for youth in Guyana? If you could do something for your community to prevent the violence occurring in adolescent relationships, what would it be?

**Source:** Rodney (2017).

**Table 2.** Summary of Interview and Focus Group Questions for Parents/Teachers/School Officials.

Adolescent dating relationships	What are your thoughts on adolescent dating?What parts of relationships and sex should be learned at home and school?How do you know adolescents are dating? (Focus Groups)
Dating violence	When you hear dating violence, what comes to mind? Why do you think dating violence occurs in Guyana? It seems like the only dating stories that make the news are those when someone is killed. Do you hear or know about any other sorts of violence in relationships? (Focus Groups)
Community awareness	What kinds of violence happen on school grounds? Does dating violence occur? Can you give me an example of a situation and how you knew? Is violence something that is talked about? If so, where and when is it mostly discussed? (Focus Groups)
Ethnicity and class	Would you say experiencing violence in Guyana is the same or different if you are Afro-Guyanese or Indo-Guyanese? (Interviews and Focus Groups) Does living in one part of Guyana as opposed to another effect being in situations that are violent? (Interviews and Focus Groups)
Gender	Are there different expectations of boys/girls in relationships in Guyana? What are they? (Focus Groups)
Prevention of dating violence in adolescence	As a parent/teacher/school official, what structures and resources do you feel would have to be in place to discuss issues such as dating violence consistently in a meaningful way in schools? If not schools, where would be an ideal place? If you could do something for your community to prevent the violence occurring in adolescent relationships, what would it be? (Focus Groups)

**Source:** Rodney (2017).

Focus group discussions were organized around two fatal dating violence stories that were covered by Guyanese newspapers. Using real-life stories to guide the discussion ensured that study participants could not deny the reality that Guyanese adolescents were dating and experienced dating violence—even if not openly discussed. By using these stories with each group, it deepened our analysis of participants’ perspectives on dating violence in relation to race, gender, sexuality, class, and age and provided opportunities to consider how these perspectives converged and/or differed within and across participant groups. One article explained the story of a 17-year-old Afro-Guyanese girl who was beaten and stabbed to death by her boyfriend after her boyfriend’s mother called her to their home to discuss cheating rumors. The second article addressed the story of a 19-year-old Indo-Guyanese girl who was stabbed to death by her alleged ex-boyfriend after an argument ensued about ending their relationship. All focus groups included a moderator and observer (Krueger & Casey, 2000), who was a Guyanese based professional with extensive experience working with adolescents in Guyana. We conducted two focus groups with each participant group which provided us further opportunity to explore topics that arose in the first group discussion. Key informant interviews with school officials and parents were completed by the first author and did not utilize the news stories used in the focus group discussions. These participants were knowledgeable about dating and dating violence and provided contextual information about these phenomena occurring on school grounds and with past students. Interviews ranged from 30 minutes to 2 hours and focus groups lasted between 1.5 hours to 2.5 hours, where participants engaged in compelling discussions and debates on the questions posed.

### Rigor and Trustworthiness

The research team engaged in reflexive practices throughout the study to ensure rigor. Individual perspectives and methodological decisions, advisory board recommendations, contextual knowledge, and qualitative research literature were discussed in this process (Merriam, 2009). Trustworthiness was achieved through source and method triangulation, by speaking with different groups of community members and utilizing interviews, focus groups, and demographic questionnaires for data generation (Merriam, 2009). A comprehensive audit trail detailed key methodological decisions, as well as the data analysis process and the researchers’ positionality (Rodney, 2017).

### Data Analysis

Data analysis was an iterative process that was guided by an intersectional lens. Analysis occurred throughout the duration of the study and became more focused once all data were collected and transcribed (Creswell, 2009; Merriam, 2009). Recordings were transcribed and transcriptions were verified against the recordings by the first author before erased. Coding was employed as a strategy to organize the data inductively and deductively using Dedoose software as a data management tool (Merriam, 2009). The authors met regularly to discuss their interpretation of transcripts and divergent meanings. The codes were expanded and reduced until a coding system was created from which the major categories and themes for this study emerged (Creswell, 2009). Memos were used to document analysis decisions and ideas as a form of analysis verification (Birks et al., 2008).

## Results

For the context of this study, adolescent dating relationships are defined as relationships between adolescent girls and boys that presumably include sexual intimacy and are not bound to specific timeframes. In terms of defining femininity, two key points were discussed by adults and youth: female respectability related to sexuality and the relationship between clothing, sexuality, and social class. Regarding defining masculinity in adolescent boys, the salient themes centered on masculinity were reproducing normative assumptions of femininity and understanding the use of violence as associated with race. Participants also identified gender roles that both men and women and adolescent boys and girls should embody, which can create opportunities for overcoming adversarial roles in heterosexual relationships.

### “Once a Girl Gets a Lot of Boys, It’s Shame:” Female Respectability and Sexuality

Participants’ perspectives revealed that women’s bodies are central to the discussion on femininity, and the ways in which adolescent girls are taught about their own bodies places them in an inequitable position entering dating relationships. Parents, teachers, and adolescents described the sexualization of girls’ bodies in their communities or girls’ sexual activity as profoundly detrimental to girls’ respectability, which is normative femininity in Guyana. As one school official stated,

They must learn that there are certain things as young ladies you don’t do to this priceless body. It is beautiful, it is clean, and the more beautiful it is, the more clean it is, the highest price you’ll get for it. (Female, Interview 6)

This participant indicated that she was not referring to financial trade or women being bought and sold, rather she was indicating that the value of women decreases if they are sexually active. Comments such as this teach girls that the value of their bodies is not dependent on their own beliefs but based on how they are perceived by others. Not only is their value measured by men who may date women, but also by other women whose ideas about girls’ worth are informed by narrowly defined perspectives on gender. In this sense, the worth of adolescent girls is measured and valued by the “purity” of their physical bodies, which means not being sexually active. This presumes that women bear responsibility for any negative experience they may encounter, if they are not perceived to be adhering to dominant discourses on femininity. Entering dating relationships with this perspective forecloses any conversation around consent and the healthy exploration of one’s sexuality as adolescents and can create an unhealthy starting point for couples to negotiate tensions and disagreements. The act of providing consent begins from a position that adolescent girls have power to decide whether they will engage in a sexual relationship. However, when adolescent girls learn that the value of their bodies is dependent on what others think, it teaches them that they have less power over decision-making about their bodies. Further, this discourse perpetuates an environment of judgment and preconceptions, fostering the silence of adolescent girls when experiencing violence.

Evidence of a sexual relationship has major consequences in some cases. For instance, both teachers and students stated that they knew girls who were sent away from their families when they became pregnant. Even if families chose to support adolescent mothers, minimal services exist to encourage young pregnant mothers to stay in school. Those that do are not a part of the formal education system and have limited capacity to support adolescent girls. Moreover, in discussions with parents, one mother told the group that she took her daughter to be checked by a gynecologist to prove her virginity after her daughter was accused of having sexual relationships. Irrespective of how unfounded the claim against her daughter was, the mother’s actions illustrate the social pressures that families experience when their daughter’s reputation is questioned. This example provides some insight into the shaming women and girls experience for being sexually active and how girls may be less likely to report partner violence because they fear disclosing sexual activity.

Discussions in the adolescent girls’ and boy’s focus groups corroborated such views. One participant in the girls’ focus group provided two examples where, from her perspective, girls encouraged boys to be disrespectful.

Boys don’t just go and take advantage of girls, just like that. They get some girls (the group interjected, saying “ya,” “true”), go and deh with Tom, Dick, and Harry [some girls have sex with multiple people]. (G2, Girls Focus Group)

If women choose to be sexually active with multiple partners, they are deemed as not respectable and, therefore, they bear the responsibility for being mistreated. However, the same participant went on to explain that if girls choose to have sex with boys who have lied to them, then they are not intelligent. Therefore, rigid ideas of respectability for girls persist within adolescent perspectives and are reinforced even within adolescent girls’ judgment of each other’s sexual actions.

Adolescent boys revealed that some of them accept normative gender discourses by acknowledging the double standard within their perspectives. The statement below illustrates that adolescent boys did not take responsibility for the degrading name-calling that reproduced judgmental and categorical statements about adolescent girls. Rather, they faulted girls for not conforming to the double standard and placed the onus on them to change their behavior. In this sense, boys did not question how their statements and actions contributed to gender inequity.

(…) I will be truthful to you. We are boys and we cannot stay with one partner, because one wood can’t light a fire (laughter in group). But for the girls that go around getting different boys, we would call them names like “dangles” and “whores” and all those types of names, but if boys do it, it would not look so bad. That is why I think the girls should conduct themselves better. (B5, Boys Focus Group)

As these examples indicate, heteronormative femininity dictates that women should either avoid sexual relationships or have a single partner to be considered respectable and deviating from these gender norms could lead to public shaming. However, not all adolescent girls agreed with this perspective. There were some girls who resisted the dominant views brought forth by the group and chose to befriend boys to understand how they think and as well as for friendship. One adolescent girl who did not conform to the rigid gender roles attributed to women spoke. She stated,

Ms., I have a lot of male friends and because of that certain people tend to criticize me. Ms. because they see me around a lot of boys and because of that they think, well, “she deh with all of dem, and she is this” [*she deh with all of them* means dating and/or having sex with all of them] Ms., but I don’t care what they have to say because I learn more from the boys than the girls. (G7, Girls Focus Group)

This teen was aware that her actions went against the dominant view of how girls should behave, but she chose to resist those ideas acknowledging that her relationship with those boys helped her to understand how they think and her interaction with them was about friendship.

### “What Is Exposed Is Expired:” Clothing, Female Sexuality, and Class

Rigid ideas of femininity produced intolerance and violence against women and girls who do not conform to the ideology of femininity and feminine respectability in Guyana. There were several examples offered by participants where girls judged other women and described how adolescent girls experience judgment, harassment, and at times violence, if they did not conform to community beliefs regarding female clothing and modesty. Of note, perspectives on clothing were also thought to reflect one’s social class, where women living in lower socioeconomic communities were described as wearing more revealing clothes and were labeled as “ghetto” in the girls’ focus group. Participants explained that there is “good and bad attraction” and the attraction women receive is dependent on the choices they make with their clothing.

These responses reflected a mindset among adolescent girls that foster a mentality of victim-blaming. Clothing style was used to judge girls’ character, even in the case of abusive relationships. If they dressed modestly, they could not be accused of “fooling around.” This was the belief of one male teacher when discussing one of the adolescent dating violence stories discussed in focus groups. He explained to the group that he did not believe the victim was in a relationship with her murderer because she was “a good Muslim girl.” When asked to expand on what this meant and how he knew this, he referred to her clothing and that she was wearing the appropriate attire that kept her covered.

However, if adolescent girls have attributed clothing and ghetto-ness to “bad attraction,” the possibility exists that the fear of being labeled as not respectable could be more important for social positioning than leaving an unhealthy relationship. Interestingly, girls expressed anger and annoyance with how boys objectified and judged them based on how their bodies looked in their clothing. Yet, they too contributed to this verbal judgment against other girls who dressed in ways that went against their ideas of respectable clothing.

But what people tend to say is what is exposed is expired. What you wear tells who you are. So, if you wear something tight or you wear something short, they will talk, like oh, she’s a freak or oh, she’s a hoe. (G4, Girls Focus Group)

Girls also explained that adolescent communities take it upon themselves to enforce clothing norms, sometimes with violence, when girls do not comply. In the quote below, an adolescent girl recounts a conversation had with other teens.

[He said] …we beat the girl last night, budday [“budday” is a slang term in Guyana referring to a friend or acquaintance] because she is a female, right? And she goes to school and she is supposed to be wearing skirt. They tell she if she don’t start wearing skirt, they gone beat she. (G4, Girls Focus Group)

However, not all girls agreed with this discourse. While participants who spoke from the dominant perspective asserted themselves as an authority and were judgmental in their tone and responses to those who challenged their thinking, three girls disagreed outright with the statements made by the majority in the group.

Hey some people? If you dress bad and look stink, people still trouble, they don’t care if you dress good, you dress fancy, people gone still get something to talk about you. (G2, Girls Focus Group)

Adolescent girls’ comments indicate that clothing is a form of social class expression and that performing respectability can also be income related. Perspectives on femininity and feminine respectability objectify women by focusing on what they wear, how they present themselves physically, and their sexual activities. Adolescent girls who live in lower socioeconomic communities and either do not want or cannot afford to conform to dominant ideas on clothing can be discriminated against due to the stereotyping of their communities.

### “Certain Girls Like a (…) Boy That Have Experience…:” Masculinity Impacted by Normative Femininity

Men’s and some women’s ideas of masculinity are heavily dependent on the complementary but subordinate roles they believe women should play in relationships. Participants revealed that dominant social scripts dictate that men should lead in relationships and this can be achieved through sexual prowess and financial support. For men who may not have the financial capacity to care for women, as is the case for many adolescents, sexual relationships create an opportunity for men and adolescent boys to fulfill their masculine roles. When women are viewed as sexually naïve in heteronormative relationships, the perception exists that girls and women require guidance. This then provides an opportunity for men to carry out the role as innate protector and leader.

Miss, certain girls like a (…) boy that have experience to guide a relationship because if none of the partner does not have experience, how will the relationship work? (B4, Boys Focus Group)

There are two main assumptions in this comment. First, if a boy is not leading a dating relationship, then neither the boy or girl has relationship experience and, secondly, that two inexperienced partners are unable to learn together about relationship roles and expectations. This creates a complex predicament particularly for adolescent girls who, according to these assumptions, must remain with little to no experience and never lead relationships. When girls do not conform to the dominant idea of female subordination, boys can struggle to find ways of affirming their manhood out of the narrow confines of masculinity and this frustration can lead to violence as another indicator of masculinity. These ideas are also present in adult relationships and can create examples for male teens that justify maintaining rigid gender roles at the detriment of adolescent girls’ physical and emotional wellbeing.

In the teachers’ focus group, a male teacher explained his interpretation of a phrase often used in Guyana, which states, “tie the heifer, loose the bull, let the bull run free.” This phrase is predominantly understood as parenting advice. The heifer refers to daughters. To tie the heifer means to keep daughters sheltered and protected by the family or home from men. The bull refers to sons who are given freedom to do as they please. If someone’s son (bull) gets to date or have sex with another person’s daughter (heifer), it means you as a parent has not kept your girl child protected. However, in the excerpt below, a male teacher provided a different interpretation of the saying by interpreting it as instructions for men who feel threatened by women who do not conform to particular notions of femininity.

What they doing is describing actually a sexual behaviour between man and woman. When it comes to the relationship, they actually telling the man what he needs to do when he meet a young lady and both of them are experienced when it comes to sex. (T1, Teacher Focus Group)

Men view sexually experienced women as a liability to their perceived inherent dominance in relationships. As this male teacher continues to speak, he indicated that a man then looks for other ways to keep the woman in a submissive state, so no other man can take her, and this can include violence. As a 10-year-old child, this same male teacher witnessed a woman beaten for not completing her responsibilities as a woman (i.e., cooking for her husband, cleaning, looking after the children). He stated,

In that case, I did not see it as violence, as how you term it … I just see it was a corrective tool to remedy the situation (laughter in the focus group). That’s what I see it as. (T1, Teacher Focus Group)

In this situation, violence was not seen as such, because of its necessity to keep women submissive, as indicated by this teacher. Witnessing a woman beaten for not completing the tasks women should be responsible for provides some insight into how understandings of violence could be shaped in relation to ideas of normative masculinity and femininity. When violence is used to assert masculinity, it is not necessarily considered violence because women have many duties and obligations to fulfill that are thought to require the supervision of men.

### Understanding the Male Use of Violence in Relation to Race and Class

When participants used race to denote differences in explaining masculinity, the similarities between ethnicities were more evident than the differences. Race was used in these contexts to sustain longstanding stereotypes. Race, in these instances, did not reveal nuances that would be helpful in meeting the needs of individual communities to promote healthy relationships between men and women. The boys’ focus group revealed that the use of violence in relationships and the understanding of masculinity was explained through their understanding of race as scientific facts. The discussion among adolescent boys illustrated that colonial ideas of race remain central in defining who they are and their understanding of masculinity and violence as innate. Interestingly, the participant who was of Mixed race and only identified his Portuguese background beside the word “mix” on his demographic form—leaving out his other racial identities—believed that he was least likely to resort to violence.

B5: Mostly Indians, Miss, they drink poison. It seems that when you are Indian, the best way is suicide.

B1: For Africans, I think they would kill their partner. They just kill. They might not. I think scientists say that East Indians are more likely to kill themselves, but the Africans would kill their partners or whatever. They would just get angry and kill out the partner, but the East Indians would just kill themselves.

B5: I agree with [B1], Miss. African people, we are just stupid (laughter). No offense, Miss, they don’t know how to control their aggression. They just want to do what their mind tells them, they don’t have a mindset. That is why most times Indian people are right, because African people, they don’t think.

B2: From the mixed perspective, they would talk around the problem. Anytime. Moderator: Who would talk about the problem?

B2: The mixed. Like a mixed person, they would talk around the situation. Work things out and they wouldn’t let anger take them over.

Participants’ perspectives provide insight into dominant discourses of patriarchy that intersect with race. In most cases, adolescents have accepted narratives that are based on long-standing stereotypical perspectives of race and perpetuate violence as an appropriate male response. The connection of violence to race has gone unquestioned which can pose a challenge to eliminating GBV. If violence is assumed to be a characteristic that is unchangeable (i.e., race), it may be used to rationalize the use of violence in relationships.

### Moving From Adversarial Gender Roles to Shared Vulnerabilities

In each focus group discussion, we engaged participants in an ice-breaker exercise inviting them to describe relationship roles for men and women as well as boys and girls. The answers were documented on a flipchart for the group to see. While most answers reflected roles consistent with heteronormative masculinity and femininity (i.e., respectively, financial provider and care of children and home), there were other attributes participants believed that both men and women should contribute to the relationship. These attributes reflected healthy emotional connections and tangible suggestions of how to display such emotions in relationships.

Parents and teachers believed that men and women should support each other, complementing each other’s strengths and weaknesses, sharing the decision-making within the relationship, being committed and faithful to each other, listening and being empathetic, and operating as a team. When asked how these roles differ for adolescents, parents focused on respecting sexual boundaries and focusing more on education.

When adolescents were asked what gender roles men and women are responsible for in relationships, their answers were similar to those provided by adults with little variation noted between the adolescent boys’ and girls’ focus groups. When adolescents were asked to focus on relationship roles in their age group, boys stated that both parties should accept each other for who they are and not what they can provide and show mutual affection in a sexual manner. Interestingly, both suggestions presented by the boy’s focus group contradicted the dominant discourse of men and boys as financial providers and sexually experienced. Adolescent girls answered similarly, however they focused on concepts such as mutual respect, trust, and helping each other to focus on schoolwork.

The identified attributes in the ice-breaker exercise that were provided by participants illustrate a different starting point for conversations on relationships that focuses on sharing the responsibilities for communication and showing affection—areas where both adolescent boys and girls feel vulnerable differently. These shared realities can potentially shift the focus from the adversarial nature of heteronormative femininity and masculinity to healthier collaborative and supportive practices.

Our results illustrate that adolescent girls and boys have learned to socialize and interact with each other in intimate relationships in complex ways. These behaviors are informed and reinforced by dominant heteronormative discourses on gender, race, class, and age that are connected to a colonial past. These ideas contribute to adversarial gender roles that are enacted in adolescent relationships in ways that contribute to violence. Some elements of intimate relationships covered in our results are not new to the discussion on violence within relationships and participants have lived out these realities at times through acts of resistance. However, the ways in which societal discourses intersect in Guyanese society provide greater understanding of the complexities that interplay to create environments that sustain systemic acts of violence.

## Discussion

This article sought to address the dearth of information on how gender roles are constituted in the everyday lives of adolescents in Guyana, how these realities can contribute to GBV and how shifting away from these normative, adversarial gendered roles can provide opportunities for healthier adolescent relationships (Contreras-Urbina et al., 2019; Mancey, 2008; Reyes et al., 2016). By including adolescents, parents, and educators we were able to obtain a multi-faceted view on how gender roles and violence interact.

Our study illustrates the importance of creating safe and open spaces to engage communities in dialogue on taboo topics. In Guyana, it is not common for adolescents to engage in open conversations on dating, sex, and violence. When these topics are approached, it is often to deter dating and sexual experiences, even though some community members are aware that adolescents are dating and sexually active (Rodney, 2017). Notwithstanding these dominant views, participants in this study spoke candidly, engaging in conversations that enabled us to move beyond GBV as an individual behavioral issue, to one that includes systemic factors.

Discussions about femininity and masculinity illustrate that the concepts of respectability and reputation are dependent on each other and do not work as dichotomous value systems as Wilson (1995) initially argued. Teenagers learn that respectability for adolescent girls is manifested through the way they dress, how much their bodies are exposed or not as well as being associated with sexual purity. Being respectable is a way to avoid “bad attraction,” that is men’s and boy’s manifesting their sexual desires towards adolescent girls. Within this notion of respectability there is a firm assumption that girls are not sexually active and as a result are at their “highest price,” preserving their reputation as being respectable for intimate relationships in adulthood.

The dominant discourse suggests the main responsibility of protecting the respectability of adolescent girls is attributed to parents. The assumption is that a respectable mother raises a respectable child, and this entails maintaining sexual abstinence, or as the Guyanese phrase states, “tie the heifer.” Given that child-rearing is often considered the responsibility of women (Rodney & Bobbili, 2019; Contreras-Urbina et al., 2019), mothers may feel greater social pressure to maintain a respectable image. The strong priority of many parents in asserting their social position in the community as parents of girls who are respectable has several consequences, creating potentially adversarial relationships between parents and teens. As the results illustrated, a mere accusation of being sexually active led a mother to verify and share information about her daughter’s virginity. It also impacts parents’ communication with their daughters, potentially silencing adolescent girls if they are experiencing GBV, as this could imply a dating relationship, which does not align with a respectable image.

Conversely, when girls attempted to resist the colonial value system of respectability, their reputation within mainstream society was threatened, resulting in possible stigmatization, and in some cases also emotional or physical abuse by other teenagers who feel entitled to enforce notions of respectability. Nonetheless, there were some girls who rejected the notions of respectability in relation to clothing and asserted that “being in style” was important because regardless of what girls wear, they can be spoken about in a negative manner within the community.

Adolescent boys revealed that they too operate in the value systems of respectability and reputation. Wilson (1995) proposed that reputation, the more egalitarian value system, was the opposite colonial value system to respectability. However, adolescent boys illustrated that their reputation as the leader in relationships is dependent on adolescent girls having no sexual or relationship experience. When girls have little to no experience in relationships, specifically regarding sexual behaviors, it perpetuates the notion that boys are needed to assume a rightful leadership position within dating relationships. Without girls playing the role of respectable women, adolescent boys are unable to secure their reputation within the community as the couple’s lead. The importance of asserting this position at all costs was also revealed by a male participant who explained a Guyanese saying that he interpreted as instructions on how to gain a dominant position within a relationship when a woman is sexually experienced. In summary, for girls, respectable becomes submissive.

In addition, adolescent boys’ reputation of being sexually experienced in the community is linked to engaging in sexual activities with many girls. While these behaviors are acceptable for adolescent boys and necessary for their “fame,” as experienced sexual partners and in charge of the relationship, it occurs at the expense of defaming girls’ reputations as “whores” and “dangles,” directly attacking their respectability in the community (Wilson, 1995). Therefore, boys’ understanding of “fame” contradicts claims that reputation represents equality, rather it reinforces traditional gendered roles where men benefit at the expense of women (Besson, 1993; Green, 2006; Wilson, 1995).

These results align with previous work on respectability and reputation in the Caribbean (Besson, 1993; Green, 2006; Wilson, 1995). Caribbean scholars have emphasized that Caribbean women’s lives did not always reflect the colonial value system of respectability and men benefitted from respectability because of their privileged position in society (Besson, 1993; Green, 2006). Green (2006) further attests that even though Wilson describes reputation as being egalitarian, he did not consider that “the male pursuit and construct of ‘reputation’ might be mediated by ideologies and practices of male supremacy and female subordination” (p. 10). Furthermore, the contemporary experiences of adolescent girls bear a familiarity to the experiences of Afro-Guyanese and Indo-Guyanese women during periods of slavery and indentureship, where violence was acceptable if Guyanese women did not conform to colonial notions of femininity (Beckles, 2003; Kempadoo, 2003).

The similarity in experiences between adolescent girls and women across different historical periods illustrates how colonial ideologies remain steadfast and pervasive in the everyday lives of people. Our work contributes to this body of knowledge by illustrating how the subordination of adolescent girls is manifested through their sexuality and clothing while adolescent boys maintain their privilege without questioning the hierarchal relationship between themselves and adolescent girls.

A focus on gender roles also revealed that adolescent boys understand the use of violence to be different based on race. This is not to imply that race does not factor into defining gender roles for adolescent girls, because Indian and African women are discursively constituted by racial stereotypes in a Caribbean context (Beckles, 2003; Kempadoo, 2003; Trotz, 2003, 2004). Rather, for participants in this study, the discussion on femininity and gender roles interestingly converged around sexual respectability across race. Colonial racist discourses are regularly used to explain violence, as illustrated by a male participant who believed that being “mixed race” means being less prone to use violence. His comments revealed that he considers mixed-race people superior to Indian or African Guyanese because lighter skin equates to greater civility. Therefore, the understanding of race remains an undercurrent in defining adversarial gender roles because it is indivisible from ideas of gender—even if it is not recognized as such.

Moreover, the link between race and adversarial gender roles is important as it continues to reveal the normalization of violence in relationships based on stereotypical understandings of an innate nature of violence in relation to race. These perspectives highlight that more effort is required to engage adolescents and their surrounding communities in discussions on race that can address and correct deeply embedded stereotypes as a form of decolonization. Our results also indicate that without questioning how the understanding of race informs ideas of masculinity, the acceptability of violence may continue because it is naturalized as a characteristic based on race. Furthermore, further research is needed to understand how adolescent girls’ experiences of violence are mediated in a society that remains polarized by racialization (Trotz, 2004).

Lastly, it should be noted that while dominant discourses supported adversarial gender roles, there were areas of resistance by some girls in focus group discussions. This illustrates that some adolescent girls are living out their truth and questioning the sensibility of conforming to gender roles that challenge the everyday realities of being an adolescent girl or young woman in Guyana. More importantly, discussions revealed opportunities that could be considered to move polarized discourses on gender norms and relationships to a common ground, because adolescent boys and girls are both vulnerable in different ways.

Adolescent boys and girls have made it clear that they are dating, they want their dating experiences to be healthier and they also desire more opportunities to discuss these topics openly. However, their experiences revealed the societal tensions that make it difficult to achieve healthier relationships. Violence prevention initiatives within this context must go beyond addressing individual behavior to account for systemic factors that contribute to the understanding of gender roles and violence. Notably, this realization must extend to parents, teachers, and adults who interact with adolescents and can influence their perspectives and experiences. Creating more opportunities for teachers, parents, and other adults to support each other in addressing these topics may better meet the needs of teenagers as an important component of violence prevention. Our work will also be useful to other populations of adolescents within Guyana, the Caribbean region, and the Caribbean diaspora to provide opportunities for comparisons and critiques as to how context is important in understanding issues such as dating violence. In this sense, our work is transferable as it maintains the meanings within our study while still being useful to consider for other contexts and situations (Creswell, 2009; Houghton et al., 2013).

## Study Limitations

While this study provides important information on heteronormative gender roles and GBV among racially diverse adolescents in Guyana, our work did not explore the complexities of interracial dating relationships and dating violence for adolescents in a Guyanese context. Additionally, the focus on heteronormative dating relationships means that our results do not include the voices of LGBT2SQ+ adolescents and the location of a secondary school in the capital city, means that we did not capture the perspectives of adolescents who live in the hinterland regions of Guyana.

## Conclusion

Discourses on normative masculinity and femininity are connected to a colonial past that must be considered in order to address GBV. The current understanding of normative masculinity and femininity created misconceptions and adversarial relationships between adolescent boys and girls where girls most often experienced an oppressed position (shame) in heterosexual relationships, exposing them to stigma and GBV, but boys’ experiences are valued (fame). Social relationships with parents and teachers can also contribute to GBV, if perspectives of adults are exclusively informed by dominant discourses on gender. Moving away from adversarial roles to shared vulnerabilities provides a healthier starting point for adolescent relationships and continues the work of decolonization with adolescents.

## Supplemental Material

Supplemental material for this article is available online. Click here for additional data file. Supplemental Material for Sex as Boys’ Fame, But Girls’ Shame: Adversarial Adolescent Gender Roles and Gender-based Violence in Guyana by Ruth Rodney, Denise Gastaldo, D. Alissa Trotz, and Claire V. Crooks, in Journal of Interpersonal Violence

## Author Biographies

**Ruth Rodney**, RN, PhD, is an Assistant Professor in the School of Nursing at York University. Her research examines adolescent dating violence, violence prevention, and healthy relationship development through community approaches accounting for systemic and structural factors. She is particularly interested in African, Caribbean, and Black populations in these regions, Canada, and globally.

**Denise Gastaldo**, RN, PhD, is an Associate Professor in the Faculty of Nursing at University of Toronto. Her research explores health as a social phenomenon. She focuses on how health is produced through social, political, and economic processes, exploring migration and gender as social determinants of health, both in Canada and internationally.

**D. Alissa Trotz**, PhD, is a Professor in the Women and Gender Studies and Caribbean Studies at University of Toronto. Her research trajectory unfolds across related themes that address processes of social reproduction, neoliberalization & Caribbean feminisms; coloniality, racial formations, gendered difference and violence; transnational migration, remittances, and diaspora engagement; and Caribbean knowledge production.

**Claire V. Crooks**, PhD, C Psych, is a Professor in the Faculty of Education at Western University and the Director of the Centre for School Mental Health. Currently, she researches dating violence and mental health promotion, evaluation of school-based mindfulness and social-emotional learning programs, and resilience programming for newcomer youth in schools and community settings.
