# Supplementary material for: Sex as Boys’ Fame, But Girls’ Shame: Adversarial Adolescent Gender Roles and Gender-based Violence in Guyana
Source: J Interpers Violence. 2021 Sep 11;37(21-22):NP19237–64. doi: 10.1177/08862605211043585 (PMC9554368; doi:10.1177/08862605211043585)
Supplement: Supplemental material for this article is available online. [file sj-pdf-1-jiv-10.1177_08862605211043585.pdf]

July 30, 2021

Dear Dr. Conte,

Thank-you for accepting our manuscript titled, *Sex as boys' fame, but girls' shame: Adversarial gender roles and gender-based violence in Guyana* for publication. We would like to thank the reviewers once again for their astute comments which have undoubtedly contributed to a stronger manuscript. We have endeavoured to make it clear how each point was addressed. We appreciate reviewer 2's interest in our work and suggestions to expand on several areas in the paper. We have attempted to address their comments while also being mindful of the manuscript length. Below we have created a chart that details all comments by reviewers and how we have addressed them.

**Reviewer 1**	
**Comments**	**Authors Response**
On page 1 "within the past 12 months" sounds like the authors are referring to the actual last twelve months rather than research describing prevalence over a prior year.<br>In the same paragraph "high rates of violence among women" reads as though women are being violent against each other – it should say "high rates of violence against women."	Thank-you for these comments. We have changed both sentences for greater clarity. Regarding the first comment, the report was describing the 12 months prior to data collection illustrating that younger women were experiencing more violence compared to older women.
I agree completely with the authors critique of the concept of 'saturation." However, they still need to justify their small sample size – they write "Parents and students from different households were recruited for focus group discussions by sending information letters home with two Form 4 (Grade 10) and two Form 5 (Grade 11) classes." Did they interview everyone who consented to the study? How many people got recruitment letters and how many responded? It is perfectly legitimate to have this small sample for this exploratory study, but the authors need to at least mention that only 5 boys and 10 girls agreed to participate in the study. If 50 boys and 100 girls agreed to participate, and only 5 boys and 10 girls were interviewed (which I know is not what happened) that would be problematic. If, on the other hand, the authors included everyone who consented,	Thank-you for this comment. We have included more information to clarify the sample size on pg. 7.<br><br>"*All participants who agreed to be interviewed or participate in a focus group were included in the study, except for the boys focus group. While eight boys agreed to participate, five showed up to focus group discussions.*"

that strengthens the credibility of the study findings.	
**Reviewer 1**	
**Comments**	**Authors Response**
perhaps provide some examples immediately here...	Thank-you for this comment. It is difficult to determine which sentence the comment is for. However, we believe this comment is in relation to the sentence ending with "*longstanding factors that contribute to the acceptability of gender-based violence*". We believe the following two paragraphs provide examples of factors that should be considered. Namely, we identify historical violence, colonial legacies within specific social institutions and the understanding of age in a Caribbean context.
Is there evidence or a history of violence between these different groupings? Does racial diversity impact on the question at hand?	Yes, there are. We have provided examples of how the understanding of race and the use of violence has been understood and passed down to adolescent boys in our manuscript. See page 18. We also discussed that even though girls did not explain experiences of violence through race, there are historical examples if these connections. See page 23.
as a marriage?	This has been changed.
This historical lens... (as opposed to 'a')?	This has been changed.
are not (as opposed to have not)?	This has been changed.
perhaps also important to note the impact of this on the (narrower) family setting... communities may not openly discuss things that families still might... but if there is silence across the board then this is particularly taboo...	Thank-you for this comment. We have edited the sentence to include your thought on the potential impact within the family. See below.<br><br>*Consequently, experiences that arise from dating relationships are not discussed openly within community settings, even if at times they are discussed within the familial home.*
in Guyana. (I know this is pedantic, but I think it is important that you continue to highlight this focus).	We have included 'Guyana' into the three suggested areas.
perhaps here you could note that this is a simple (and simplistic) understanding of gender roles, that pervades patriarchal (and heteronormative) societies globally.	This has been included. See page 3.
Given the explicit focus on parenting and child-rearing, it is unclear...	This has been added.
add, "elsewhere around the globe"	This has been added.

do they attempt to explain this? If they do, perhaps speak to this explanation briefly here...	Thank-you for this comment. The authors do explain it and the references are provided should the reader want further information. However, for the purposes of our literature review, we have highlighted the point of the paper that is important.
I think the authors should say something about the tension between protector and predator. Even if it is just too highlight it...	We provided these articles to illustrate another view in the literature regarding the relation of gender-roles and the connection to violence. While we appreciate the reviewer's point, we have chosen to forego this change.
where some research has...	This has been changed.
other research finds that	This has not been changed as it is the same study. See edit on pg. 5.
but are you talking to gendered assumptions here? The primary difference identified here seems to be the gender of the person, not the assumptions they hold. If the assumptions are tied to the gender, then this is something that needs to be made clearer.	Yes, we are speaking about gender and assumptions. In the original passage we indicate that adolescent boys had strong attitudes accepting of violence. However, we have changed it to provide greater clarity. See pg. 4-5.<br><br>*…where some research has found that adolescent girls were not more susceptible to accepting physical dating violence even though they had been exposed to familial violence, while boys who had experienced childhood familial victimization had strong attitudes accepting physical violence against women and girls (Debowska et al., 2017). Therefore, the impact of violence on youth can vary based on gender and gendered assumptions (Debowska et al., 2017; Ulloa et al., 2008)*
there are words missing here... as it stands this doesn't make sense...	Please see edit on pg 5. Excerpt included below.<br><br>*International literature on gender-based violence reveal that the understanding of traditional gender norms in relation to the use of violence must be addressed, yet there are gaps in our knowledge.*
therefore also bearing significant responsibility in terms of the socialisation of the Guyanese youth into gendered life... this is implied, but make it explicit.	Please see edit on pg 6. Excerpt included below.<br><br>*They are informed by interactions with the communities that surround adolescents and therefore bear significant responsibility for the socialization of Guyanese youth into gendered life*
reverse order 'it is' to 'is it'...	This has been changed.
perhaps acknowledge how small this number is... perhaps given that there were only 2 of them you could also indicate	We had planned to complete 5 interviews in total, including two parents as key informant interviews to the school environment where the study took place. We mention later in the paragraph that we did not

whether they were the parents of one family or two...	include two people from the same household in the study.
perhaps mention here, given your small sample size, the total number of potential participants that this yields...	Reviewer 1 also provided similar feedback and we provided further information to indicate that those who consented to participate were included in this study.
does this include non-heterosexual relationships?	The newspaper examples used to facilitate discussion in the focus groups were heterosexual relationships and youth did not discuss same-sex partnerships.
if you are only talking about heterosexual relationships then this must be made clear	Please see edit below to the sentence. Pg. 10<br><br>*Participants also identified gender roles that both men and women and adolescent boys and girls should embody, which can create opportunities for overcoming adversarial roles in heterosexual relationships.*
In my context, there are cultures that include something called a bride-price or lobola. Is this kind of practice captured under financial trade? Because I think members of these cultures would consider it a misunderstanding of the practice to think of it as buying and selling girls and women...	Thank-you for this insight. The practice you have indicated is not what this participant was referring to or would be captured under financial trade. We asked the participant to explain her comments and that was her response. We have edited the sentence to make it clear that it was the participant's comment and not our interpretation. See below.<br><br>*This participant indicated that she was not referring to financial trade or women being bought and sold, rather she was indicating that the value of women decreases if they are sexually active.*
ideas about a girl's worth	This has been changed.
or are not perceived as adhering?	This has been changed.
replace 'are' with 'is' here	This has been changed.
I don't see why this follows... Autonomy is classically taken to refer to the freedom or control that a person has over their actions (or what moves them to action)... do you mean to say that their autonomy is diminished when it comes to controlling the meaning that their bodies have in the eyes of others? I think this may simply require rewording, so that what you want to express is expressed.	Please see the edit below.<br><br>*However, when adolescent girls learn that the value of their bodies is dependent on what others think, it teaches them that they have less power over decision-making about their bodies.*
replace with 'heteronormative'?	This has been changed.
deviating from	This has been changed.

Perhaps say a bit more about this. Where do these so-called 'deviant' ideas come from?	We believe the example provided is sufficient to explain why some young girls choose to go against the dominant perspective.
Perhaps emphasise that frustration in this regard could lead to violence (as another indicator of masculinity)?	This has been added. See edit below.<br><br>*When girls do not conform to the dominant idea of female subordination, boys can struggle to find ways of affirming their manhood out of the narrow confines of masculinity and this frustration can lead to violence as another indicator of masculinity.*
yes this is a fascinating section, which could still be expanded upon.	Thank-you. We believe it is interesting as well. However, the paper is already quite lengthy (over 9000 words) and we believe what we have presented achieves the aim for the overall paper.
heteronormative	This has been changed.
I would have liked to have seen more on this...	We believe the sentence before illustrates a divergence from the dominant beliefs of boys/men's roles in relationships.
this could be expressed with more clarity...	See edit below.<br><br>*Some elements of intimate relationships covered in our results are not new to the discussion on violence within relationships and participants have lived out these realities at times through acts of resistance*
you have not actually discussed this at all yet, though... neither have you come back to the idea of vulnerability mentioned at the outset of the paper (which I recall played a role in this shift)...	Thank-you for this comment. However, we discussed this in the results section titled, "Moving from adversarial gender roles to shared vulnerabilities" on pg. 19-20.
as well as being associated with sexual purity (in relation to their actions, not simply (or only) the way they dress -- the way they dress is read as an indication of the kinds of activities they engage in)	See edit below.<br><br>*Teenagers learn that respectability for adolescent girls is manifested through the way they dress, how much their bodies are exposed or not as well as being associated with sexual purity.*
this is not yet included in the discussion. It is only now coming up. You should either fix this by moving the discussion of vulnerability up into the findings, or making it clear above that this discussion is only engaged with here at the end of the paper.	Thank-you. We have moved a paragraph from the discussion into the results to further expand on the results section titled, "Moving from adversarial gender roles to shared vulnerabilities" on pg. 19-20.
